# Comparing Public Attitudes to Traditional Biomedical Versus Trauma-Informed Approaches in Psychosis Services

**DOI:** 10.1093/schizbullopen/sgag014

**Published:** 2026-04-23

**Authors:** Joshua Kearns, Gavin Davidson, Ciarán Shannon, Ciaran Mulholland, Donncha Hanna

**Affiliations:** School of Social Sciences, Education and Social Work, Queen's University Belfast; School of Social Sciences, Education and Social Work, Queen's University Belfast; Northern Health and Social Care Trust, Holywell Hospital, Northern Ireland, United Kingdom; Northern Health and Social Care Trust, Holywell Hospital, Northern Ireland, United Kingdom; School of Medicine, Dentistry and Biomedical Sciences, Queen’s University Belfast; School of Psychology, Queen's University Belfast

**Keywords:** early psychosis, psychosis, stigma, trauma-informed, mental health attitudes, mental health services

## Abstract

**Objective:**

Stigma is a significant barrier to engagement with psychosis services. This study explored whether trauma-informed mental health services are perceived as less stigmatizing and more effective than traditional biomedical services, and examined individual-level predictors of such perceptions.

**Methods:**

A cross-sectional online survey was conducted with 1460 participants via Amazon Mechanical Turk. Respondents were randomly assigned to read one of two vignettes describing either a trauma-informed or traditional biomedical psychosis service. Attitudes toward the service were measured using service-related attitudinal questions grounded in the tripartite framework Olson and Zanna (*Annu Rev Psychol*. 1993;44:117–154) and informed by stakeholder perspectives. Multivariate Analysis of Variance (MANOVA) compared differences across service-model vignettes. Exploratory factor analysis identified two latent factors, perceived stigma and service effectiveness, which were used as regression outcomes to determine individual predictors. Four hierarchical regressions investigated predictors of mental health attitudes and knowledge, adverse childhood experiences (ACEs), and social desirability, and their impact on stigma and service effectiveness.

**Results:**

Trauma-informed services were rated significantly less stigmatizing and more effective across all dimensions (*P* < .001, η^2^ = .02–.10). Perceived stigma was strongly associated with positive mental health attitudes, greater ACE exposure, and liberal political orientation. Notably, mental health knowledge was linked to higher stigma, suggesting that factual understanding alone may not reduce negative perceptions.

**Conclusions:**

Trauma-informed services are perceived as less stigmatizing and more effective than traditional biomedical models. Findings highlight the influence of individual attitudes and experiences on stigma, supporting contact-based, context-aware public campaigns to improve perceptions of psychosis and promote compassionate, trauma-informed mental health care.

## Introduction

Over the past number of decades, research into psychosis and other psychotic illnesses has increasingly focused on the causal impact of trauma. It has been well documented that childhood trauma is a major risk factor for many mental health disorders such as depression, anxiety, and bipolar.^1^ Psychosis is no different, and studies have consistently demonstrated that adverse childhood experiences (ACEs) are strongly associated with an increased risk of psychosis.^2,3^ However, emerging population-based evidence suggests that higher ACE exposure is associated with more negative perceptions of mental health services, potentially driven by poorer early experiences of care, reduced trust in professionals, and increased discomfort engaging with healthcare systems, supporting calls for trauma-informed approaches in healthcare settings.^4^

Previously, the traditional biomedical model has dominated care for individuals across many mental health conditions, including psychosis, prior to the insurgence of psychosocial collaboration with service users. This model would routinely include pharmacological interventions at the core, with a focus on biological causes for mental health disorders, often characterizing such illnesses as “Brain Diseases.”^5^ This model has been important in the development of treatment options and encouraged the growth of evidence-based psychological treatments and helped thousands across the world. However, this biological framework has not come without “side-effects”; people with psychosis can experience negative outcomes including increased stigmatization, decreased trustworthiness, and disempowerment due this traditional perspective.^5,6^ This points to an obvious gap between knowledge and stigma, highlighting the limitations of a purely biomedical lens in addressing the lived and relational aspects of psychosis.

Trauma-Informed Care (TIC) has emerged as a paradigmatic shift that repositions psychosis services within a framework of safety, collaboration, and interpersonal context.^7^ Recent work has been used to capture the essence of trauma-informed practice through the lens of professional and clinician perspectives via Delphi method. These perspectives have pinpointed the principles of TIC in Early Intervention Services (EIS) for Psychosis.^8^ Sixteen identified principles cover domains such as empowerment, trustworthiness, and trauma knowledge as essential components, which form the basis for TIC within the EIS. These approaches have tentatively shown improvements in treatment adherence, attendance, and outcomes,^9,10^ potentially due to the non-judgmental and less stigmatizing ethos.

While clinical perspectives are essential, limited research has examined public views on psychosis and trauma. Existing evidence, however, consistently shows that the general public holds more stigmatizing attitudes and beliefs toward psychosis compared to other mental health conditions like depression or anxiety.^11^ These attitudes may be amplified according to political orientation, with right-wing authoritarian ideology associated with more negative attitudes, likely mediated by moral judgments, perceived threat, and beliefs about personal responsibility.^12^

Evidence for the effectiveness of public campaigns to raise awareness and reduce stigma, especially over the long term, is limited.^13^ The Lancet Commission reported that awareness alone is insufficient and reinforced that direct social contact with lived experience is essential.^14^ Further, a review of mental illness stigma hypothesized that negative public perception was largely due to the mainstream consensus of mental disorders being attributed to biogenic causes.^15,16^ Consequently, different causal perspectives can affect treatment attitudes. For example, Lüllmann et al.^17^ found in a general population sample that when schizophrenia is framed as biologically caused, people report less motivation for treatment and feel less personal control over symptoms. Similarly, qualitative studies show that individuals with psychosis often criticize the biological model for emphasizing medication over other therapeutic approaches.^18^

Further research is therefore needed to better understand stigma about psychosis, at individual, services, and societal levels, and how to address it.

### Research Aims

To our knowledge, this is the first study to examine public perceptions of trauma-informed services in comparison to traditional biomedical psychosis services. The research had three primary aims: first, to examine whether trauma-informed services differ from traditional biomedical services across service-related attitudinal items; second, to evaluate the underlying structure of these items using factor analysis; and third, to identify factors associated with perceived stigma and perceived service effectiveness. These factors included mental health attitudes, demographic variables, mental health knowledge, and personal experiences of trauma.

## Methods

### Study Design and Participants

A quantitative, cross-sectional survey design was employed. Participants were recruited via Amazon Mechanical Turk (MTurk), a widely utilized online platform for data collection in psychological research. Eligibility criteria included a minimum 95% approval rating on previous MTurk tasks and residency in the United States. Participants received $4 for completing the study, which was estimated to take ~20 min, aligning with fair compensation standards.

### Procedure

Upon accessing the study through MTurk, participants were redirected to the Pavlovia platform, where they were randomly assigned to one of two groups:

Group 1: Questionnaire accompanied by a traditional biomedical service vignette.

Group 2: Questionnaire accompanied by a trauma-informed vignette.

Data were collected via Pavlovia and exported for analysis. Incomplete submissions (*N* = 1) were excluded. To ensure data quality, three attention-check questions were embedded within the questionnaire (e.g., “Select ‘Strongly Agree’ if you are still paying attention”). Participants who failed any attention check were excluded from the analysis (*N* = 91). Final data were analyzed using SPSS version 27.

### Ethical Considerations

Ethical approval was obtained from the Queen’s University Belfast School of Social Sciences, Education and Social Work. Participants provided informed consent prior to participation.

### Measures

#### Demographic Information

Participants provided demographic information including age, gender, employment status, ethnicity, education level, and political ideology (See Table 1).

**Table 1:** Demographic Composition Comparing Traditional Biomedical versus Trauma-Informed Service Vignettes

Demographic	Traditional biomedical (*N* = 725)	%	Trauma-informed (*N* = 735)	%	χ^2^	*P*
**Gender**						
Male	423	50	429	50	.99	.80
Female	298	50	301	50		
Non-binary/third gender	4	50	4	50		
Prefer not to say	0	0	1	100		
**Age group**						
18-24	19	46	22	54	1.9	.93
25-34	318	51	311	49		
35-44	251	49	259	51		
45-54	64	49	68	51		
55-64	47	52	44	48		
65+	26	46	30	54		
Prefer not to say	0	0	1	100		
**Race/ethnicity**						
White	644	50	643	50	3.1	.79
Black	24	48	26	52		
Hispanic	11	44	14	56		
Asian	29	45	36	55		
Native American	13	59	9	41		
Two or more ethnicities	2	29	5	71		
Prefer not to answer	2	50	2	50		
**Education level**						
Less than high school	0	0	2	100	8.6	.28
High school graduate	36	55	30	46		
Some college	50	50	51	50		
Associate’s degree	32	41	47	59		
Bachelor’s	450	50	457	50		
Master’s degree	150	52	136	48		
Doctoral degree	7	41	10	59		
Prefer not to say	0	0	2	100		
**Political opinion**						
Very liberal (far-left)	42	44	53	56	4.3	.63
Liberal	140	47	157	53		
Moderate	133	49	136	51		
Conservative	174	50	176	50		
No opinion/not political	7	58	5	42		
Prefer not to answer	6	67	3	33		

Percentages reflect row distributions. χ^2^ = chi-square test statistic; *P* = significance level.

#### Community Attitudes to Mental Illness Scale

The Community Attitudes to Mental Illness Scale (CAMI^19^) is a 40-item scale designed to assess public attitudes toward mental illness. It comprises four subscales: Authoritarianism, Benevolence, Social Restrictiveness, and Community Mental Health Ideology. Responses were rated on a 5-point Likert scale, with higher scores reflect more positive attitudes toward mental illness. The CAMI demonstrates adequate internal consistency for the total scale and acceptable test–retest, supporting its use as a valid tool for assessing attitudes toward mental illness.^20^

#### Mental Health Knowledge Schedule

The Mental Health Knowledge Schedule (MAKS^21^) is a 6-item scale used to measure mental health knowledge as a component of stigma. Items were rated on a 5-point Likert scale, with higher scores indicating greater mental health knowledge. The MAKS shows acceptable internal consistency, test–retest reliability and validity supported by expert review, for assessing stigma-related mental health knowledge.^21,22^

#### Adverse Childhood Experiences Questionnaire

The Adverse Childhood Experiences Questionnaire (ACE-Q^23^) retrospectively measures exposure to adverse experiences before the age of 18. It includes ten binary (yes/no) items covering experiences such as physical, emotional, and sexual abuse. The ACE-Q has demonstrated good internal consistency^23-25^ and is widely used in both research and clinical settings to reliably quantify childhood adversity and its impact on health outcomes.

#### Vignette Scenarios

Two vignette scenarios were developed to represent a fictional service user engaging with either a trauma-informed mental health service or a traditional biomedical service. Participants were randomly assigned to view one of the two vignettes. The scenarios were co-produced with input from individuals with lived experience, clinicians, and researchers. The initial narratives were grounded in real-life accounts shared by individuals with lived experience. These were then modified to ensure structural equivalence across vignettes, isolating key differences between trauma-informed and traditional service models. Consensus on content and fidelity was established through consultation with clinicians and researchers working in the EIS, including psychologists and psychiatrists. Further refinements were made to enhance accuracy and ensure a balanced and valid comparison between conditions (see Supplementary Section 1).

#### Stigma Associated with Service

Following the vignette, participants responded to a series of statements assessing perceptions of stigma and service effectiveness associated with the service described. These items were developed in accordance with Olson and Zanna’s^26^ tripartite model of attitudes (affect, behavior, cognition). These newly developed items were informed by consultation with researchers, clinicians, and individuals working in the EIS to capture both internalized and externalized stigma, as well as assessing comparison of perceived service user effectiveness. All eight items were newly developed for this study and were designed to capture participants’ perceptions of how the service and its users may be viewed, rather than respondents’ own personal stigma. As is common in stigma research,^27^ several items were designed to operationalize social distance in order to enhance ecological and face validity. For example, statements such as “I would feel uncomfortable if someone I knew used this service” were included. Responses were measured on a 5-point Likert scale ranging from Strongly Disagree to Strongly Agree (see Table 2 for full list of questions).

**Table 2:** ANOVAs Comparing Agreement Ratings to Stigma Questions across Services

Question	Traditional biomedical		Trauma-informed		*F*	*df*	*P*	**η**_**p**_^**2**^
	Mean	SD	Mean	SD				
Olivia may feel anxious or upset about revisiting this service.	4.01	0.90	3.56	1.08	74.87	1, 1458	< .001	0.05
Olivia may feel responsible for her condition, due to the opinions of the clinician.	3.61	0.99	3.09	1.26	76.44	1, 1458	< .001	0.05
I would feel uncomfortable if someone I knew used this service.	3.07	1.35	2.67	1.43	30.53	1, 1458	< .001	0.02
Olivia will feel more able to explore her trauma.	3.23	1.20	3.87	0.95	126.65	1, 1458	< .001	0.08
Olivia will find difficulty in being accepted in her community after attending this service.	3.43	1.09	3.01	1.19	48.45	1, 1458	< .001	0.03
I would avoid recommending this service to a friend or family member.	3.20	1.23	2.66	1.39	61.07	1, 1458	< .001	0.04
This service provides an open and clear channel for Olivia to give her thoughts on her illness and explore her previous trauma.	3.34	1.28	4.09	0.93	166.15	1, 1458	< .001	0.10
I believe this is an effective service for meeting all of Olivia’s mental health needs.	3.33	1.22	4.02	0.94	148.49	1, 1458	< .001	0.09

#### Marlowe–Crowne Social Desirability Scale—Short Form

The 13-item Marlowe–Crowne Social Desirability Scale (MD-SDS^28^) Short Form was used to assess the tendency toward socially desirable responding. Items were scored as either “True” or “False.” Higher total scores indicated a greater likelihood of social desirability bias. The MD-SDS Short Form exhibits high internal consistency reliability and correlates strongly with the original 33-item scale, validating it as a psychometrically sound and efficient measure of social desirability bias.^28,29^

## Results

### Statistical Analysis

Data analysis was undertaken in three stages. The first stage examined differences across service-related attitudinal items between trauma-informed and traditional biomedical services. A MANOVA and separate univariate tests were used to examine differences on the same eight questions following service vignettes. Stage 2 determined the latent variables of these eight questions using a factor analysis. This allowed for regressions in stage 3 to be conducted in determining the factors associated with lower levels of stigma.

In conducting regression analyses, the categorical variable gender was coded as 1 for “male” and 2 for “female”; a reference category was not made for other gender identities due to a small sample size (*n* = 4, <0.01%). Liberal was dummy coded as 1 for Liberal vs Moderate and Conservative, and vice versa for Conservative vs Liberal and Moderate. There were no significant differences between vignette groups on mental health knowledge, ACE scores, or political orientation (all *P* > .05).

### Main Outcomes

#### RA 1: Examine Differences between Trauma-Informed and Traditional Biomedical Services across Service-Related Attitudinal Items

To determine differences between trauma-informed versus traditional services, a MANOVA was completed according to eight service-related attitudinal questions following vignette scenarios. Using Pillai’s Trace = .167, *F*(8, 1451) = 36.247, *P* < .001, partial η^2^ = .167, a significant moderate to large sized effect of type of service on attitudes was evident. Subsequently, separate univariate ANOVAs were used to compare agreement levels by service vignette on each of the eight attitudinal variables. A Holm–Bonferroni correction^30^ was applied to adjust for multiple comparisons.

When the service was described as trauma informed rather than traditional, there was significantly greater agreement that the service would allow users to feel less anxious about revisiting the services; service users feel less responsible for their condition due to clinician attitudes and feel more able to explore trauma; and the service would provide a more open and clear channel for users to express thoughts and experiences. Participants were less likely to feel discomfort if someone they knew accessed the trauma-informed service; they were less inclined to believe users would face community stigma following service use; and they were more willing to recommend the trauma-informed service to friends or family members. Additionally, there was greater agreement that the trauma-informed vignette more effectively met all of the user’s mental health needs. Partial eta-squared values for all effects ranged from .02 to .10, representing small to medium effect sizes (see Table 2).

#### RA 2: Explore the Underlying Factor Structure of Attitudinal Items Using Factor Analysis to Identify Distinct Dimensions of Service-Related Stigma

To determine the suitability of the attitudinal items for exploratory factor analysis (EFA), separate analyses were conducted for each of the two vignettes. For the traditional biomedical vignette, the Kaiser–Meyer–Olkin measure of sampling adequacy was .724, and for the trauma-informed vignette, it was .799, both exceeding the recommended threshold of .60, indicating sufficient sampling adequacy.^31^ Bartlett’s Test of Sphericity was also significant for both traditional (χ^2^ = 1770.28, *df* = 28, *P* < .001) and trauma-informed (χ^2^ = 1999.93, *df* = 28, *P* < .001) groups, suggesting that correlations between items were sufficiently large for factor analysis.^32^

Two factors emerged consistently across both groups. In the traditional biomedical vignette, Factor 1 (Perceived Stigma) had an eigenvalue of 2.21 and accounted for 27.6% of the variance, while Factor 2 (Perceived Effectiveness) had an eigenvalue of 2.58 and accounted for 32.26% of the variance. In the trauma-informed vignette, Factor 1 (Perceived Stigma) had a higher eigenvalue of 3.34, accounting for 41.71% of the variance, and Factor 2 (Perceived Effectiveness) had an eigenvalue of 1.64, accounting for 20.53% of the variance (see Table 3 for loadings across vignettes).

**Table 3:** Stigma Questions following Vignettes, Showing Factor Loadings and Internal Consistency of Factors from Exploratory Factor Analysis

Question	Traditional biomedical vignette		Trauma-informed vignette	
	Perceived stigma	Service effectiveness	Perceived stigma	Service effectiveness
Olivia may feel anxious or upset about revisiting this service.				
Olivia may feel responsible for her condition, due to the opinions of the clinician.	.696		.79	
I would feel uncomfortable if someone I knew used this service.	.721		.825	
Olivia will feel more able to explore her trauma.		.85		.797
Olivia will find difficulty in being accepted in her community after attending this service.	.734		.81	
I would avoid recommending this service to a friend or family member.	.796		.841	
This service provides an open and clear channel for Olivia to give her thoughts on her illness and explore her previous trauma.		.846		.796
I believe this is an effective service for meeting all of Olivia’s mental health needs.		.874		.837
Reliability (α)	.848	.74	.738	.845

Extraction method: principal axis factoring. Rotation method: Oblimin. Only loadings >.6 reported.

Although the sample size was sufficiently large, a loading cut-off of .60 was used to ensure strong statistical contribution and interpretability of items, consistent with the recommendations of Guadagnoli and Velicer^33^ and Field.^34^ The item “Olivia may feel anxious or upset about revisiting this service” did not meet the loading threshold of .60 for either factor and was removed based on statistical criteria. Items 2, 3, 5, and 6 loaded onto factor 1, and items 4, 7, and 8 loaded onto factor 2 (see Table 3). The resulting factors, Perceived Stigma and Effectiveness, were summed to create composite scores used in subsequent regression analyses.

#### RA 3: Identify Key Factors Associated with Lower Levels of Perceived Stigma Associated with the Service Described, Including Participant Characteristics and Attitudes

Four separate hierarchical regressions were conducted to assess two factors of perceived stigma and service effectiveness for participants who either completed traditional biomedical or trauma-informed service vignettes.

### Perceived Stigma

In the traditional biomedical service condition, the final model explained 31.5% of the variance in stigma, *F*(8,699) = 41.67, *P* < .001. The inclusion of social desirability in the initial step accounted for a small proportion of variance (∆*R*^2^ = 1%). The addition of demographic variables such as age, gender, and political orientation contributed a further 12% of explained variance, with older age and female gender associated with lower stigma, and conservative political orientation linked to slightly higher stigma. Liberal orientation was not statistically significant. Introducing mental health attitudes and knowledge improved the model substantially, adding an additional 16% of explained variance. More positive mental health attitudes were strongly associated with lower stigma, while higher mental health knowledge showed a positive association with perceived stigma scores. In the final step, inclusion of ACE scores accounted for a further 4% of variance, with higher ACE exposure associated with higher stigma levels. Notably, the effect of social desirability diminished in the full model, indicating that stigma ratings were relatively robust to self-presentation bias.

In the trauma-informed condition, the final model explained 49.3% of the variance in stigma. Social desirability initially accounted for a similarly small share (∆*R*^2^ = 1%), while demographic and political orientation variables explained 23% of the variance when added. As in the traditional biomedical condition, older age and female gender were linked to lower stigma, and conservative orientation to higher stigma. The third block, comprising mental health attitudes and knowledge, accounted for an additional 23% of variance, with attitudes showing a particularly strong negative association with stigma. ACE scores again contributed a modest but meaningful increase of 4% in explained variance. Overall, although the same variables were significant in both models, the trauma-informed service condition yielded greater explanatory power, particularly for attitudinal variables.

### Perceived Service Effectiveness

In the traditional biomedical vignette model, the final regression explained 28% of the variance in perceived effectiveness. Social desirability alone accounted for 1% of the variance. The inclusion of demographic and political variables increased this by 5%, though gender and age had weaker effects and political orientation was not a significant contributor. Mental health attitudes and knowledge added a substantial 20% of explained variance with more positive mental health attitudes negatively associated with perceived effectiveness. However, knowledge was positively associated with perceived service effectiveness. ACE scores contributed an additional 3% in the final step. Altogether, the model showed that individuals with greater knowledge and higher ACE scores perceived the traditional biomedical service as effective. In contrast, more positive mental health attitudes were associated with lower perceived effectiveness.

Comparatively, the trauma-informed model accounted for only 16.7% of the variance in perceived effectiveness. Social desirability explained negligible variance, and the addition of demographic and political orientation variables increased explained variance by 6%. In contrast to the traditional biomedical condition, age and liberal orientation were significant in this model, with older and more liberal participants the trauma-informed service as effective. Mental health attitudes and knowledge contributed an additional 11% of variance, with both variables remaining positively associated. The inclusion of ACE scores added only 1%. Despite overlapping significant variables, the trauma-informed model had lower explanatory power overall for perceived service effectiveness.

Across both conditions, mental health attitudes, knowledge, and ACE scores were the most consistent and significant predictors of both stigma and effectiveness perceptions. For both vignette groups and factors, unstandardized regression weights, coefficient standard errors, standardized regression weights, and *R*^2^ change values, by step and contributing variable are presented in Tables 4 and 5.

**Table 4:** Summary of Traditional Biomedical and Trauma-Informed Hierarchal Regressions for Variables Contributing to Perceived Stigma

Variable	*B*	SE *B*	β	**∆*R*** ^**2**^	*B*	SE *B*	β	**∆*R*** ^**2**^
	Traditional biomedical service				Trauma-Informed service			
**Step 1**				**.01**				**.01**
Social desirability	−0.17	0.08	−0.08^*^		−0.24	0.10	−0.09^*^	
**Step 2**				**.12**				**.23**
Social desirability	−0.11	0.08	−0.05		−0.14	0.09	−0.05	
Gender	−1.09	0.26	−0.15^***^		−1.38	0.30	−0.16^***^	
Age	−0.71	0.12	−0.22^***^		−1.20	0.13	−0.30^***^	
Liberal	0.39	0.39	0.05		−0.66	0.43	−0.07	
Conservative	1.46	0.34	0.21^***^		1.95	0.39	0.22^***^	
**Step 3**				**.16**				**.23**
Social desirability	−0.05	0.07	−0.02		−0.05	0.08	−0.02	
Gender	−0.79	0.24	−0.11^***^		−0.75	0.25	−0.08^***^	
Age	−0.36	0.11	−0.11^***^		−0.51	0.12	−0.13^***^	
Liberal	0.54	0.35	0.07		0.46	0.37	0.05	
Conservative	0.69	0.31	0.10^*^		1.02	0.33	0.12^***^	
Attitudes to MH	−0.08	0.01	−0.48^***^		−0.12	0.01	−0.64^***^	
MH knowledge	0.36	0.05	0.29^***^		0.36	0.05	0.22^***^	
**Step 4**				**.04**				**.04**
Social desirability	−0.01	0.07	−0.01		−0.04	0.08	−0.02	
Gender	−0.69	0.23	−0.10^***^		−0.65	0.24	−0.07^**^	
Age	−0.32	0.11	−0.10^***^		−0.48	0.12	−0.12^***^	
Liberal	0.36	0.34	0.04		0.19	0.36	0.02	
Conservative	0.43	0.31	0.06		0.64	0.325	0.07^*^	
Attitudes to MH	−0.06	0.01	−0.39^***^		−0.11	0.01	−0.56^***^	
MH knowledge	0.33	0.04	0.26^***^		0.30	0.05	0.18^***^	
ACE score	0.26	0.04	0.22^***^		0.30	0.04	0.21^***^	

^*^ *P* < .05. ^**^P < .01. ^***^P < .001. ACE, adverse childhood experience; MH, mental health.

**Table 5:** Summary of Traditional Biomedical and Trauma-Informed Hierarchal Regressions for Variables Contributing to Perceived Service Effectiveness

Variable	*B*	SE *B*	β	**∆*R*** ^**2**^	*B*	SE *B*	β	**∆*R*** ^**2**^
	Traditional biomedical service				Trauma-Informed service			
**Step 1**				**.01**				**.00**
Social desirability	−0.23	0.08	−0.12^**^		0.016	0.054	0.011	
**Step 2**				**.05**				**.06**
Social desirability	−0.20	0.07	−0.10^**^		−0.00	0.05	−0.00	
Gender	−0.53	0.25	−0.08^*^		−0.01	0.17	−0.00	
Age	−0.44	0.11	−0.15^***^		0.4	0.08	0.19^***^	
Liberal	−0.45	0.37	−0.06		0.87	0.25	0.17^***^	
Conservative	0.23	0.32	0.04		0.27	0.23	0.06	
**Step 3**				**.20**				**.11**
Social desirability	−0.14	0.07	−0.07^*^		0.03	0.05	0.02	
Gender	−0.21	0.22	−0.03		0.01	0.16	0.00	
Age	−0.08	0.10	−0.03		0.26	0.08	0.13^***^	
Liberal	−0.29	0.33	−0.04		0.54	0.24	0.11^*^	
Conservative	−0.56	0.29	−0.09		0.26	0.22	0.06	
Attitudes to MH	−0.08	0.01	−0.54^***^		0.01	0.01	0.07	
MH knowledge	0.37	0.04	0.32^***^		0.26	0.03	0.30^***^	
**Step 4**				**.03**				**.01**
Social desirability	−0.11	0.065	−0.05		0.03	0.05	0.02	
Gender	−0.13	0.216	−0.02		0.03	0.16	0.01	
Age	−0.04	0.101	−0.01		0.27	0.08	0.13^***^	
Liberal	−0.45	0.322	−0.06		0.47	0.24	0.09^*^	
Conservative	−0.786	0.286	−0.121^**^		0.168	0.217	0.037	
Attitudes to MH	−0.067	0.006	−0.45^***^		0.011	0.005	0.107^*^	
MH knowledge	0.334	0.041	0.293^***^		0.242	0.034	0.283^***^	
ACE score	0.222	0.039	0.205^***^		0.075	0.028	0.099^**^	

^*^ *P* < .05. ^**^P < .01. ^***^P < .001. ACE, adverse childhood experience; MH, mental health.

## Discussion

This study aimed to examine whether trauma-informed services are perceived as less stigmatizing than traditional biomedical services and identified factors associated with lower perceived stigma toward the service. In response to the first research aim, results from MANOVA and separate univariate tests indicated that participants expressed significantly more positive attitudes across all eight attitudinal items when responding to a trauma-informed vignette compared to a traditional biomedical service vignette. This supports the growing body of literature suggesting that trauma-informed frameworks foster more compassionate, person-centered perceptions of care^8,35,36^ and are less likely to be associated with blame, judgment, or perceived weakness.^5,6^ We found the largest effect sizes for “able to explore trauma” and “feeling this service meets all mental health needs”. This indicates that trauma-informed services are perceived as effective in facilitating trauma exploration safely and holistically, aligning with established principles of TIC.^37^ Conversely, the smallest effects emerged for “I would feel uncomfortable if someone I knew attended this service”, possibly reflecting enduring public stigma and social discomfort surrounding psychosis.^38^ Alternatively, this response might indicate the presence of empathetic concern, wherein individuals experience emotional difficulty at the thought of someone close to them requiring such services. Regardless of interpretation, trauma-informed services were consistently perceived as more effective, suggesting that their value is recognized even in contexts marked by discomfort and emotional ambivalence. Moreover, small effect sizes were seen in “difficulty in being accepted in community”. While there was a significant difference between traditional biomedical and trauma-informed services, the low effect size may be due to persistent externalized stigma. These findings underscore the need for trauma-informed EIS to incorporate targeted strategies for stigma reduction and community reintegration, including supported employment, family interventions, and public education campaigns, to improve social outcomes for this population.^39^ Such campaigns for psychosis should be contact based, targeted, local, and credible.^14,40,41^

Subsequent regression analyses clarified predictors of perceived stigma in both models. The trauma-informed stigma model explained substantially more variance than the traditional biomedical model, suggesting that stigma toward trauma-informed services is not only lower on average but also more influenced by the included variables. In both models, positive mental health attitudes predicted lower stigma, supporting previous findings,^42-44^ and reinforces the need for effective interventions that improve knowledge and attitudes. Consistent with recent research using anxiety and schizophrenia vignettes,^45^ older age also predicted reduced stigma. In contrast, greater mental health knowledge (higher MAKS scores) and more ACEs were associated with higher stigma. The consistent positive relationship between ACE scores and stigma across vignettes may reflect heightened sensitivity or internalized stigma among those with trauma histories, driven by disidentification from similarly affected groups and increased dissociation in response to perceived trauma stigma.^46^ Moreover, the counterintuitive effect of increased knowledge may suggest that factual understanding alone may not reduce stigma, as found by consistent stigmatizing beliefs from healthcare professionals.^47^ Corrigan and Nieweglowski^48^ argue that enhanced knowledge has limited impact on reducing stigma and instead advocate for contact-based interventions that emphasize individuals’ humanity to counter stigmatizing attitudes. This supports the evidence base that when shown ACE context, public stigmatic attitudes lower.^49^

Turning to perceptions of service effectiveness, the findings present a more nuanced picture. Despite trauma-informed services being perceived as more effective on average, particularly by individuals with more positive mental health attitudes, the regression model for trauma-informed effectiveness accounted for considerably less variance than the traditional biomedical model. This indicates that while trauma-informed services resonate with participants on a values-based level, perceptions of their effectiveness may be influenced by factors not captured in the current model, such as trust and/or previous experiences with mental health services.^50,51^ In contrast, perceptions of traditional biomedical service effectiveness were more consistently predicted by variables such as mental health knowledge and ACE scores. Both factors were stronger predictors in the traditional biomedical model, with higher MAKS and ACE scores associated with greater perceived service effectiveness across vignettes. This could likely be as a result of the difference in variance across vignettes for service effectiveness. On the other hand, more positive attitudes to mental illness (CAMI) were negatively associated with traditional biomedical service effectiveness, but positively associated with trauma-informed service effectiveness, showing further support that these services are generally viewed as more positive.^52,53^ Interestingly, age only emerged as a significant positive predictor in the trauma-informed model, showing older individuals within this sample perceived this service as more effective.

### Limitations

Several limitations should be noted. First, the vignettes employed a single, female character. Research suggests that stigma is often more pronounced toward male individuals with psychosis, particularly in relation to perceived dangerousness in schizophrenia.^54^ Future studies should explore gender variation to capture potential differences. Second, the sample was recruited through Amazon Mechanical Turk (MTurk), an online platform broadly comparable to other convenience sampling methods in terms of data reliability and research outcomes.^55,56^ The demographic composition of this study was similar to that of other MTurk studies.^57^ However, like all online recruitment methods, MTurk is subject to potential systematic biases such as self-selection and may underrepresent ethnic minority groups while overrepresenting individuals with higher education levels, potentially limiting the generalizability of findings.^57,58^ Finally, the stigma items created to assess attitudes were developed specifically for this study and, although informed by the model of attitudes and stakeholder perspective, they do not constitute a validated stigma scale. While this presents a limitation in terms of measurement, it is important to recognize that this study addresses novel research questions for which no established, validated tools currently exist. Therefore, these bespoke measures were necessary to capture specific attitudes toward trauma-informed versus traditional biomedical services. Nonetheless, further psychometric evaluation of this scale is recommended.

## Conclusion

In summary, this study is the first to compare public perceptions of trauma-informed versus traditional biomedical psychosis services in relation to stigma and perceived effectiveness. We found trauma-informed services were perceived as less stigmatizing and perceived as more effective than traditional biomedical services. Positive mental health attitudes, knowledge, and ACE scores consistently predicted both stigma and perceived effectiveness. The trauma-informed stigma model explained more variance, suggesting that these perceptions are more influenced by individual factors. While traditional biomedical service effectiveness was better predicted by included variables, perceptions of trauma-informed effectiveness may depend on unmeasured experiential or relational factors. Overall, findings support positive public perception of trauma-informed care and the need for interventions that go beyond knowledge to reduce stigma.

### Future Directions

Future research should focus on individuals who attend trauma-informed services to better understand how they perceive stigma from others and the role of relational or experiential factors in shaping effectiveness perceptions. We also call for a renewal of public mental illness stigma campaigns, particularly those targeting psychosis, to incorporate the context of ACEs in individuals with mental illness.

## Supplementary Material

sgag014_Supplementary_data

## Data Availability

The datasets generated and analyzed during the current study are available from the corresponding author on reasonable request.
