# Supplementary material for: Comparing Public Attitudes to Traditional Biomedical Versus Trauma-Informed Approaches in Psychosis Services
Source: Schizophr Bull Open. 2026 Apr 23;7(1):sgag014. doi: 10.1093/schizbullopen/sgag014 (PMC13213821; doi:10.1093/schizbullopen/sgag014)
Supplement: sgag014_Supplementary_data [file sgag014_supplementary_data.docx]

**Supplementary data**

**Supplementary Section 1. Vignette scenarios describing trauma-informed and traditional biomedical psychosis services used in the study.**

**Scenario 1: Traditional biomedical service for Psychosis**

Olivia, a 19-year-old university student, begins to experience overt psychotic symptoms, including hearing voices telling her to harm herself. She feels frightened and is encouraged by her family to visit a mental health service. This is a challenge for Olivia, as she has a fixed, false belief that the medical industry is out to get her personally.

Nonetheless, her family make an appointment and Olivia pushes herself to go. Upon entering the service for the first time, Olivia is greeted by a receptionist who asks her to fill out a standardised symptom checklist. This is a questionnaire that evaluates a range of psychological problems and symptoms Olivia then meets her psychiatrist, Dr. Harvey, who focuses on her current symptoms, asking a series of questions about her hallucinations and other psychotic experiences.

Following this brief discussion, Dr. Harvey insists on a treatment plan that focuses primarily on medication. He prescribes a powerful antipsychotic, explaining that it may cause side effects but reassuring Olivia that she will be monitored. Dr. Harvey emphasises the importance of medication compliance for managing her condition.

Several weeks later, Olivia meets with another mental health professional. When Olivia opens up about the shame she feels attending the service, the mental health professional briefly acknowledges this but continues to focus on her psychotic symptoms as the main issue. The mental health professional advises that, like any other physical illness, strict adherence to medication is crucial in treating her psychosis.

**Scenario 2: Trauma-Informed Service for Psychosis**

Olivia, a 19-year-old university student, begins to experience overt psychotic symptoms, including hearing voices telling her to harm herself. She feels frightened and is encouraged by her family to visit a mental health service. This is a challenge for Olivia, as she has a fixed, false belief that the medical industry is out to get her personally.

Upon entering the service, Olivia is warmly greeted by a receptionist who welcomes her with a calm and compassionate tone. She reassures Olivia to share her experiences in her own words. Olivia then meets Dr. Harvey, who listens attentively to her fears and concerns, acknowledging how traumatic the experience can be.

Dr. Harvey takes time to explain various treatment options, ensuring Olivia understands and has a say in her treatment plan. He emphasises that it's a collaborative effort and encourages Olivia to express her preferences. When discussing medication, Dr. Harvey explains potential side effects and assures Olivia she'll be closely monitored and will have choices about her medication.

Several weeks later, Olivia meets with a mental health professional. When Olivia shares her feelings of shame about attending the service and the distress caused by her symptoms, the mental health professional actively listens and validates her experiences. They explore how Olivia's past experiences might be related to her current symptoms, taking an integrated approach, considering multiple factors and perspectives to understand her psychosis.

The mental health professional helps Olivia understand that her psychotic experiences might be ways of coping with or adapting to past traumas, rather than symptoms of an illness. Together, they work on developing coping strategies and exploring underlying issues, empowering Olivia in her recovery journey.
